# Optimization of compression ratio in LHR engine fueled with nano Al_2_O_3_-emulsified biodiesel using RSM and machine learning

**DOI:** 10.1039/d5ra06361b

**Published:** 2025-12-19

**Authors:** Radha Krishna Gopidesi, Kautkar Nitin Uttamrao, Channa Keshava Naik N., Premkartikkumar S R, Ahmed Adnan Hadi, K. Sunil Kumar, T. M. Yunus Khan, Abdul Saddique Shaik, Ahmed A. Alamiery

**Affiliations:** a School of Engineering, Department of Mechanical Engineering, Presidency University Bengalore India gopidesi.radhakrishna@presidencyuniversity.in; b SVERI's College of Engineering Pandharpur 413304 Maharashtra India nitin.kautkar@gmail.com; c Department of Mechanical Engineering, BGS College of Engineering and Technology Bangalore 560086 Karnataka India naikphd.sit@gmail.com; d Department of Automotive Engineering, SMEC, Vellore Institute of Technology (VIT) Vellore India premgreentech46@gmail.com; e Artificial Intelligence Sciences Department, College of Sciences, Al-Mustaqbal University 51001 Babil Iraq ahmed.adnan@uomus.edu.iq; f Department of Marine Engineering, Faculty of Engineering, Sri Venkateswara College of Engineering Pennalur Chennai 602117 India sunilkumarkresearcher@gmail.com; g Department of Mechanical Engineering, College of Engineering, King Khalid University Abha 61421 Saudi Arabia yunus.tatagar@gmail.com ashaik@kku.edu.sa; h Al-Ayen Scientific Research Center, Al-Ayen Iraqi University, AUIQ An Nasiriyah, P.O. Box: 64004 Thi Qar Iraq dr.ahmed1975@gmail.com

## Abstract

This research explores the influence of different compression ratios (CRs) on the performance and emission properties of a fly ash-coated low heat rejection (LHR) diesel engine operated with a nano-Al_2_O_3_-based emulsified cotton seed biodiesel blend (B20W10Al200). An extensive experimental design was implemented based on the history data-based response surface methodology (RSM), taking brake power (BP) and CR as significant variables. Engine responses like brake thermal efficiency (BTE), brake specific fuel consumption (BSFC), and significant exhaust emissions (NOx, HC, CO, and smoke opacity) were examined over CR values of 16, 17, and 18. The findings identified CR18 as the best configuration, where the maximum BTE (29.03%) and minimum BSFC (0.269 kg kW^−1^ h^−1^) were obtained. A notable decrease in emissions was seen, most notably in CO (0.104%) and smoke opacity (19.3%), with NOx emissions significantly lower for CR16. To improve the predictive performance and facilitate optimization, machine learning methods were incorporated. Extreme gradient boosting (XGBoost) models performed efficiently, with *R*^2^ values greater than 0.90 for all the parameters. SHapley additive exPlanations (SHAP) revealed that brake power is the dominant control factor influencing the prediction of the response variable. Multi-response desirability-based optimization, performed through the Design-Expert software, indicated an optimum setup (maximize BTE/minimize BSFC and smoke while controlling NOx) at BP = 2.49 kW and CR = 18, which had a composite desirability value of 0.751. This study confirms the combined potential of thermal barrier-coated LHR engines and nano-emulsified biofuels under optimal conditions, validating the shift toward cleaner and more efficient combustion in compression ignition engines.

## Abstract 2

This study examines how varying compression ratios affect the performance and emissions of a fly ash-coated low heat rejection diesel engine fueled with a nano-Al_2_O_3_-based emulsified cottonseed biodiesel blend (B20W10Al200).

## Graphical abstract

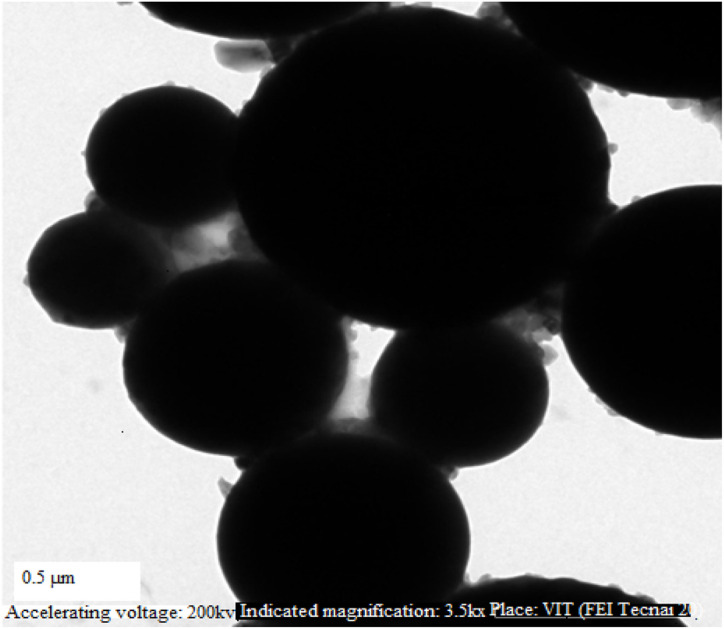

## 1. Introduction

The automotive sector constantly seeks innovative approaches to enhance the environmental sustainability and efficiency of engines. LHR engines have become popular as they have the capability to enhance the overall performance and reduce the heat-related losses.^1^ In LHR engines, their elements of cylinder liner, pistons, and valves are coated with insulating substances such as fly ash to modify the heat dissipation. Another trend that is emerging is the introduction of emulsified fuels, where hydrocarbon fuel is mixed with water, to maximize combustion and reduce pollutant emissions.^2^ The introduction of nanomaterial additives, like Al_2_O_3_, in emulsified fuels has been found to improve their combustion characteristics and engine efficiency. Compression ratio (CR) is a parameter that has a significant impact on the engine design and performance. In an LHR engine coated with fly ash, the complex interrelationship among compression ratio, fuel emulsion mixture, and nanomaterial additives forms a difficult but potentially revolutionary direction for maximizing the combustion.^3^ Biodiesel is a promising fatty alkyl monoester obtained from natural resources such as plant- and animal-based oils.^4^ Among the list of alternative fuels, biodiesel is one of the most trusted and effective alternatives according to previous research. There are many positive points regarding biodiesel, such as its spotless and organic energy sources, which works as the best option to replace petroleum-based fuels.^5^ Additionally, biodiesel can be used in regular diesel engines without any modifications, and the performance of these engines is not hampered when biodiesel is used as an alternative fuel.^6^ Consumers continuously demand petroleum products, resulting in an increase to 240 million metric tons from 2021 to 2022. If this demand continues, this number can increase to 465 million metric tons by 2031 or 2032.^7^ Most importantly, as an alternative fuel, biodiesel is better owing to its fewer emissions and negative effects than fuel combustion. Furthermore, it possesses a low sulfur particle content and high flash point and is compostable.^8^

### 1.1. Nanoparticle requirement

Moreover, some aspects of biodiesel limit its use, such as its high density, poor atomization quality, and sometimes improper combustion due to continuous use of fuel injectors, suffering carbon deposition on their surface.^9^ Notably, with higher blend levels, the performance and emission aspects of biodiesel become mediocre, similar to that of neat diesel fuel, resulting in an increase in viscosity.^10^ As technology advances daily, nanotechnology has become a rapidly growing sector. As result of this continuous improvement in nanotechnology, useful and effective nanoparticles have been introduced in several fields.^11^ Researchers have found the potential of nanoparticles as additives to enhance the thermophysical properties of fuel. A greater surface area to volume can be achieved by introducing nanoparticles in fuel. In addition, this leads to high thermal conductivity and mass diffusivity.^12^ Besides, nanoparticles as an additive in fuel can work as a catalyst, resulting in effective and efficient fuel combustion.^13^ These nanoparticles are quite valuable for biodiesel as they improve the combustion rate and overcome the issue of high viscosity. By investigating the different aspects of LHR engines and emulsified and nonmaterial additives, a number of possibilities has been found to address the current problems encountered with the use of biodiesel in an effective way.^14^

Among the available options, nanoparticle additives are also the most favorable fuel catalysts, which help to shorten the evaporation and ignition delay time at the same time. The foremost requirement of nanoparticles is listed below. Overall, the interference of nanoparticles in fuel ultimately leads to enhanced oxidation power to improve rapid fuel combustion. They should maintain the conventional engine working operation throughout their involvement. Although nanoparticles are mixed with fuel, they should remain chemically stable for better and smoother fuel combustion operation. Optimization *via* machine learning^15^ and Response Surface Methodology (RSM) can be significantly applied for the design of biodiesel engines.^16^ These approaches provide advanced means for enhancing the efficiency, implementation, and environmental impact of biodiesel engines. A vital application is engine performance prediction and optimization. With different input parameters, ML models can be trained to forecast multiple factors like fuel efficiency, emissions, and overall engine health.^17^ Concurrently, RSM provides a disciplined approach for test design, modelling, and optimization, enabling researchers to identify optimal input variable combinations for enhanced performances.

In addition, these methods are crucial elements of emission-cutting initiatives. ML models could evaluate intricate correlations among biodiesel blends, engine operating conditions, and emissions. The findings may be employed to build successful emission-reduction measures.^18^ Simultaneously, RSM optimization aids in determining the best biodiesel blend to reduce emissions, while preserving or boosting the engine performance. Due to the synergy between ML and RSM, a holistic strategy for tackling both efficiency and environmental concerns is provided.^19^

LHR was applied in an engine using a ceramic coating with oxide on parts such as the crankshaft, chambers, and fittings at a thickness of 300 µm, which did not affect the physical size of the engine parts. Petroleum diesel was combined with 20% mahua biofuel and 5% ethanol. For comparison, the combustion capacity was investigated utilizing regular gasoline and contrasted with the biofuels using a combination of LHR and LTC methodologies. Lastly, the combination of LHR and LTC improved the combustion efficiency by up to 3.48%.^20^ The mixtures are run in a naturally inhaled, steady-state combustion ignition (CI) cylinder. Yttria stabilized zirconium (YSZ) was applied to the engine crowns of the instrumented cylinder to transform it into a heat rejecting (LHR) engine. Operating the motor on antioxidant-doped JME in LHR mode increased the thermal rate of release and highest cylinder pressures by approximately 4% and 7%, respectively, at the greatest load. The percentages of CO and unaltered HC discharges declined to an acceptable level of 10% and 13%, respectively, at the highest load performance; additionally, NO pollutants were reduced by 13% at the highest load level. The mileage and energy efficiency of the LHR engine increased by approximately 7% and 11%, respectively, when fully loaded.^21^ This investigation used two pistons around both the untreated and the additional polished one. The additional engine was coated with 300 µm-thick ZrO_2_ and 6–8 wt% Y_2_O_3_ ceramic substance, which is known as YSZ. A combination of *Jatropha*, also methyl alcohol (JME), and oil in proportions of 20% and 80% was created (JME20) and utilized as pilot fuel, while oxy-hydrogen (HHO) gases served as induction fuel for dual-fuel operations. HHO gas is free of greenhouse gases and a hydrogen-based sustainable fuel. The findings demonstrated that the effectiveness exhibited by the YSZ-coated pistons during the two dual-fuel activities (D100 + HHO and JME20 + HHO) was approximately 5.5% and 5.9% greater than that of D100 operating at the highest load, respectively. The equivalent dual-fuel processes resulted in a decreased level of HC, CO, and soot, regardless of the engine capacity.^22^ Experiments were conducted at peak load at an identical FIP (600 bar) with various FIT (19, 21, 23, 25, and 27 °BTDC) and fuel mixtures (D100, JOBD20, P10JD90, P20JD80, and P30JD70). In the beginning, the tests were conducted using solely diesel at the conventional injection rate and time. Furthermore, tests were performed by exchanging just diesel with extremely high responsiveness fuel (JOBD) at various infusion schedules, while less reactive fuels were delivered *via* the inlet pipe at equilibrium of 2 bar in various quantities. All quaternary fuel procedures with injections inclinations greater than 24°BTDC resulted in an increase in the piston pressure (89.82 bar), indicating better combustion with JOBD as HRF. Lower BTE levels (4.6%) were created because the electric production by the engine was reduced at 27°bTDC injecting, causing its pressure to be fairly elevated.^23^

### 1.2. Machine learning (ML) and response surface methodology (RSM) for biodiesel application

ML and RSM are used in a large number of applications. Another field where ML and RSM are useful is fuel blend optimization. ML models can assess the impact of various biodiesel blends on engine performance, discovering the most effective combinations under given conditions. At the same time, RSM optimization guides the determination of the right mix proportions, considering properties such as viscosity, ignition properties, and energy value. This combined effort ensures that the biodiesel blend fulfils the performance requirements and aligns with environmental and operational needs. The combination of ML and RSM provides a comprehensive method for biodiesel engine research, offering forecasting techniques, optimizing and maintaining the engine performance, and addressing environmental issues. Recently, several researchers have adopted this combined approach of nanoparticle-enhanced biodiesel-diesel blends to enhance the efficiency and minimize emissions successfully. Table 1 presents a clear overview of studies that applied up-to-date machine learning (ML) and Response Surface Methodology (RSM) methods to optimize the engine performance. These studies underscore the synergistic benefit of pairing the high predictive accuracy of ML with the high optimization capabilities of RSM to enhance the engine efficiency and lower emissions.

**Table 1:** Summary of the studies using ML and RSM methods for forecasting and improving engine responses

Ref.	Techniques	Parameters	Biodiesel	Remarks
24	Artificial neural network	Performance and emission parameters	Waste cooking oil	The ANN model demonstrated exceptional predictive accuracy across all engine output parameters, with correlation coefficients (*r*) exceeding 0.99 and *R*^2^ values surpassing 0.98 for every variable
25	ANN-ANFIS, RSM	Methanol molar ratio, catalyst amount, reaction time	Neem and castor	The ANFIS model outperformed the ANN in predicting yield, exhibiting higher *R*^2^ values, and thus superior forecasting accuracy
26	ANN, RSM	Split injection parameter	Ammonia-biodiesel	The ANN model achieved *R*^2^ values greater than 0.99 for all responses, demonstrating exceptional real-time predictive accuracy and outperforming RSM in reproducibility
27	ANFIS-NSGA-II and RSM	Engine load, biodiesel blend, and nanoparticle concentration	Leachate blends with nano-additives	The ANFIS-NSGA-II model produced responses with higher accuracy and efficiency than those generated by other models
28	ANFIS and RSM	EGT and all types of emissions	Nano diesel blended fuels	The test results closely align with the ANFIS predictions, demonstrating a high level of predictive accuracy
29	DTR, ABR, ETR, GBR, LGBM, and XGBR	Engine load, compression ratio, blend ratio	*Aloe vera* biodiesel with MWCNT nanoparticles	The XGBR model achieves the highest prediction accuracy compared to all other models
30	Decision tree and RSM	CR, injection time, injection pressure	Biogas-biodiesel blends	The decision tree-based models exhibited strong robustness, characterized by low mean squared errors
31	RSM	Load and compression ratios	*Cassia fistula* and *Ricinus communis*	RSM achieved correlation coefficients (*R*^2^) between 0.92 and 0.99 for all output parameters, demonstrating high predictive accuracy
32	RSM, gradient boosting (GBoost), extreme learning machine (ELM)	BP, LCV, blends	*Moringa oleifera* biodiesel with 1-hexanol and Zr_2_O_3_ nanoparticles	The ELM model achieved the highest accuracy (*R*^2^ = 0.9604), surpassing all other models
33	AMT ML and multi-objective optimization RSM	Varying engine torque, speed	Sunflower oil	The AWOA exhibits superior precision and a faster convergence rate compared to PSO
34	RSM with desirability	Engine load, biodiesel blend, and nanoparticle concentration	Mahua with CuO nanoparticles	RSM identified M20 with 60 ppm nanoparticle concentration at 80% load as the optimal condition (desirability score: 0.9), with the model attaining a mean absolute percentage error (MAPE) of just 3%

Table 1 presents a comprehensive overview of the studies that have employed advanced machine learning (ML) and Response Surface Methodology (RSM) techniques to optimize the engine performance. These investigations highlight the synergistic advantages of combining the high predictive accuracy of ML with the robust optimization abilities of RSM to improve the engine efficiency and reduce emissions.

This study advances internal combustion (IC) engine technology through the study of a new blend of emulsified fuels, nano-Al_2_O_3_ additive, and compression ratio changes in a fly ash-coated LHR engine. It highlights the need for emission reduction *via* optimized combustion processes, a critical step towards compliance with strict environmental legislation and ensuring sustainable engine designs. The investigation of the synergy between emulsified fuels and nano-Al_2_O_3_ additives improves the current knowledge of innovative fuel technologies and provides a basis for more efficient, environmentally friendly fuel compositions. However, although the Response Surface Methodology (RSM) provides systematic and organized optimization, it seldom succeeds in identifying complex nonlinear relationships. Most existing research depended either on RSM or machine learning (ML) separately, thereby losing the chance to leverage their complementary benefits. Thus, this work fills this gap by integrating RSM with sophisticated ML models, like XGBoost, to improve the prediction accuracy and optimize the engine performance concurrently. The ensuing hybrid method presents a strong, scalable solution for optimizing biodiesel engines in accordance with worldwide sustainability objectives.

The combined application of RSM and XGBoost not only reflects methodological creativity but also yields practical knowledge regarding engine design and operational refinement. These results are a useful reference for engineers, researchers, and practitioners in the industry who want to enhance the performance and efficiency of IC engines. The capacity to successfully explore the high-dimensional parameter space of emulsified fuel-supplemented LHR engines reflects the power of advanced optimization methods in practical applications.

Improving the performance of IC engines, which is integral in a range of industries, is still key to realizing energy efficiency and environmental sustainability. The use of nano-Al_2_O_3_ additives and emulsified fuel in a fly ash-coated LHR engine represents a novel yet demanding research problem. Mechanistic appreciation of the intricate interactions between emulsified fuel formulations, nano-Al_2_O_3_ loading, and compression ratio variations is important for maximizing the combustion efficiency and reducing emissions. Therefore, this research is aimed at determining the best configurations and parameters that will ensure maximum efficiency in LHR engines through the employment of nano-enhanced emulsified fuels, and hence the development of the following research objectives.

(a) To investigate the impacts of varying CR on the combustion efficiency, power generation, and emissions in an LHR engine with a fly ash coating using emulsified fuel with a nano-Al_2_O_3_ additive.

(b) To study the impact of different nano-Al_2_O_3_ concentrations in emulsified fuel on the combustion behaviour, ignition characteristics, and engine performance as a whole.

(c) To utilize RSM to systematically optimize the compression ratio to achieve the maximum BTE considering the intricate interactions amongst the compression ratio, emulsified fuel composition, and nano-Al_2_O_3_ concentration.

(d) Using XGBoost modeling to forecast engine parameters and performance results as a function of compression ratio, emulsified fuel characteristics, and nano-Al_2_O_3_ concentration provides an integrated system understanding.

## 2. Material and methods

The selection of materials and methods plays a critical role in ensuring reliable outcomes in any experimental investigation. In this study, the use of cotton seed oil as a biodiesel feedstock was strategically chosen due to its abundant availability as an agricultural residue and its non-edible nature, making it an ideal candidate for sustainable fuel production. Cotton seed biodiesel possesses a high cetane number, ample oxygen content, and a safe flash point, which collectively contribute to improved combustion efficiency and reduced harmful emissions. Moreover, its inherent lubricating properties support engine durability by minimizing wear, thereby extending the engine life.^35^

**Fig. 1:**
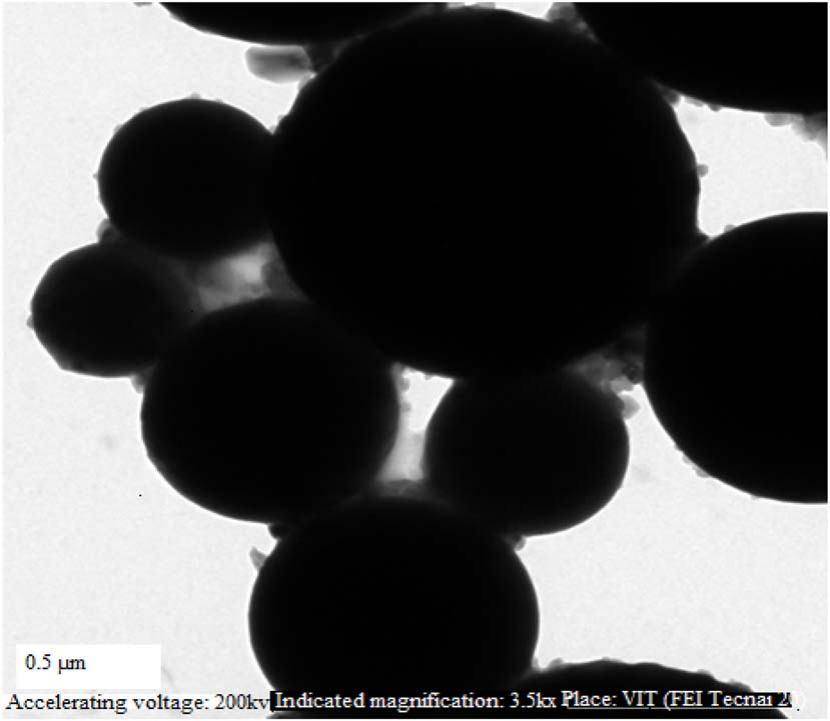
Micrograph of a sample of fly ash obtained using scanning electron microscopy (SEM).

The choice of materials and techniques is an important factor for ensuring consistent results in any experimental study. For this research, the employment of cotton seed oil as a biodiesel feedstock was selected as a deliberate choice because of its prevalent availability as an agricultural waste and its inedibility, making it a suitable material for the production of sustainable fuel. Cotton seed biodiesel has a high cetane number, high oxygen content, and a safe flash point, which together lead to more efficient combustion and less harmful emissions. Additionally, its native lubricating attributes enhance engine longevity through the reduction of wear, thus longer engine life.

From a larger viewpoint, the use of cotton seed oil not only supports waste valorization and rural economic development but also fuels sustainable energy culture by minimizing reliance on fossil fuels. The compatibility of the biodiesel with nano-additives like aluminium oxide (Al_2_O_3_) adds to its usefulness, as the nanoscale particles facilitate purification of combustion quality, reduced emissions, and optimized engine performance. Therefore, the blend of cotton seed biodiesel and nano-additives is an attractive, environmentally friendly option for improving the efficiency and sustainability of compression ignition engines. Fig. 1 illustrates Micrograph of sample of fly ash Scanning Electron Microscope (SEM).

The application of thermal barrier coatings, such as fly ash, to LHR engines helps to improve their thermal efficiency by minimizing the heat loss. LHR engines have higher in-cylinder temperatures, which promote efficient combustion, because they retain more heat in their combustion chamber.^36^ This is especially beneficial for biodiesel blends, which have lower calorific values and higher viscosities. LHR engines also reduce the ignition delay, specific fuel consumption, and hydrocarbon and carbon monoxide emissions. Moreover, the application of fly ash coatings improves the engine durability under thermal stress, making LHR engines suitable for nano-enhanced biodiesel applications for cleaner and more efficient operation.^37^

**Fig. 2:**
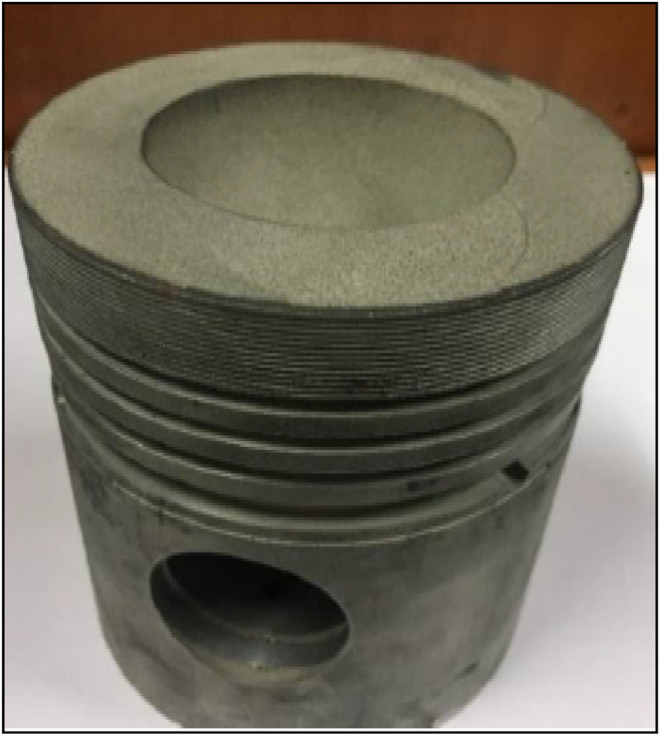
Fly ash powder-coated piston crown.

**Fig. 3:**
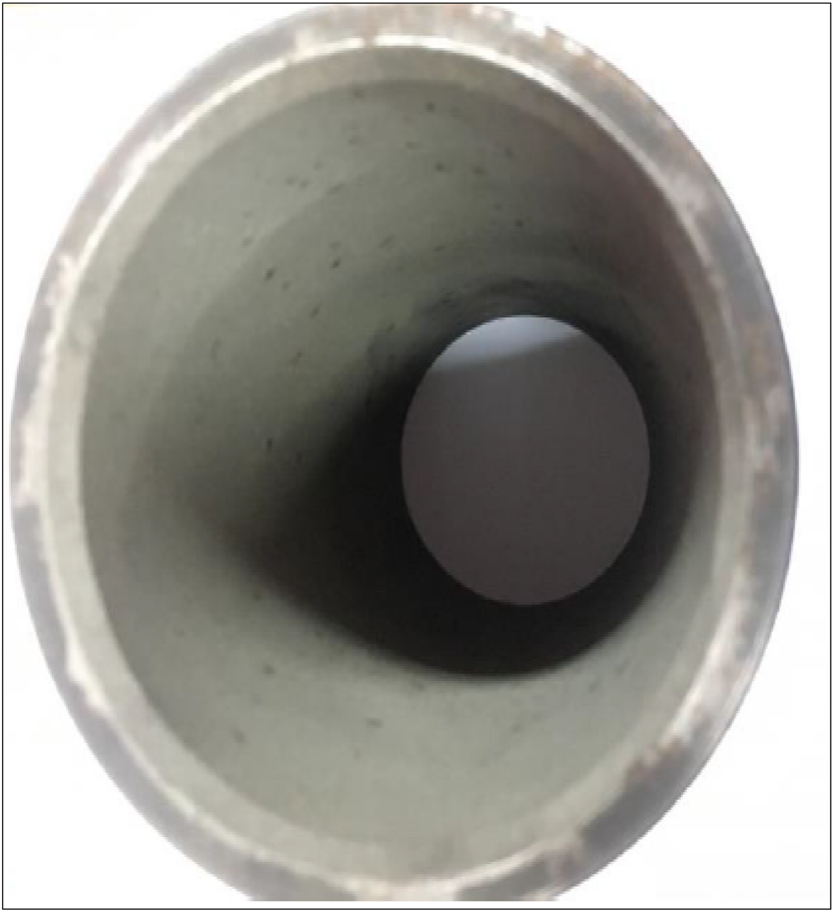
Fly ash powder-coated cylinder liner.

LHR engines are a progressive type of engine with numerous possibilities to explore with the help of fruitful experimentation work. Following this, the piston crown and cylinder liner were coated with a thickness of 200 µm fly ash as an insulating material, as illustrated in Fig. 2 and 3. To maintain a standard compression ratio (CR) before coating, this section will deal with an important step that follows a systematic strategy.

### 2.1. Preparation of nano-emulsified fuel

**Fig. 4:**
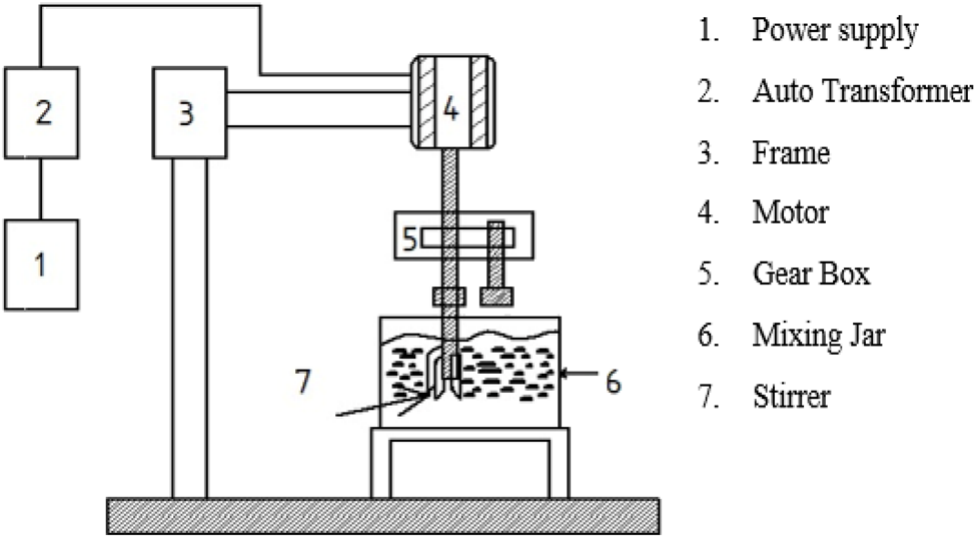
Layout of the mechanical stirrer arrangement for the preparation of the nano-emulsified fuel.^35^

In this research, cottonseed biodiesel was considered and employed. NaOH was used as a catalyst in the transesterification process for biodiesel production. Fig. 4 depicts the experimental setup for synthesizing nano-emulsified biodiesel with a mechanical stirrer.^38^ In addition, an ultrasonic stirrer was employed in the process for the preparation of the nano emulsion. As part of the nano-emulsified fuel testing groundwork, 10% water and 20% cottonseed biodiesel were mainly blended in 88% by volume, and this proportion was transferred to a vessel.

The mixture was mixed well by agitating at 500 rpm. Throughout the agitation process, a surfactant such as Span 80 and Tween 80 (2% by volume) was mixed dropwise into the biodiesel water emulsion. The HLB value for the surfactant was considered 6.34 for this preparation. The mixture of biodiesel, water, and surfactant was kept under stirring for 40 min. According to earlier studies, if the water content in diesel fuel is greater than 10%, it prolongs the ignition delay. This results in uneven engine operation, and thus 10% water was used with biodiesel for emulsion. The detailed properties of the diesel and biodiesel along with the blends and nanoparticles are presented in Table 2. By adjusting the amounts of nanomaterial addition like mass fraction of Al_2_O_3_ in B20W10, several testing fuel samples were created. Here, the Al_2_O_3_ nanoparticle concentration reached 200 ppm.

**Table 2:** Properties of the fuels and their blends

Property	Diesel	CSME	B20	B50	B20 + 100 ppm	ASTM
Viscosity (Cs)	3.35	4.68	4.042	4.56	4.726	ASTM D445
Calorific value (kJ kg^−1^)	42 858	39 528	39 496	39 484	42 058	ASTM 240
Density (kg m^−3^)	840	868	828	850	843	ASTM D1298
Flash point (°C)	84	180	174	177	175	ASTM 93
Fire point (°C)	94	123	96	86	84	ASTM 93

The B20W10Al200 test fuel sample was created using an R-4C ultrasonicator. The ultrasonicator was run at 50 to 60 kHz frequency for 40 min to achieve the ideal emulsification. The color of the equipped nano-emulsified fuel sample is milky white due to the chemical reaction between the fuel and surfactant employed in this preparation process. However, this color did not negatively impact the performance aspects during testing in the engine operation.

## 3. Experimental set-up and procedure

**Fig. 5:**
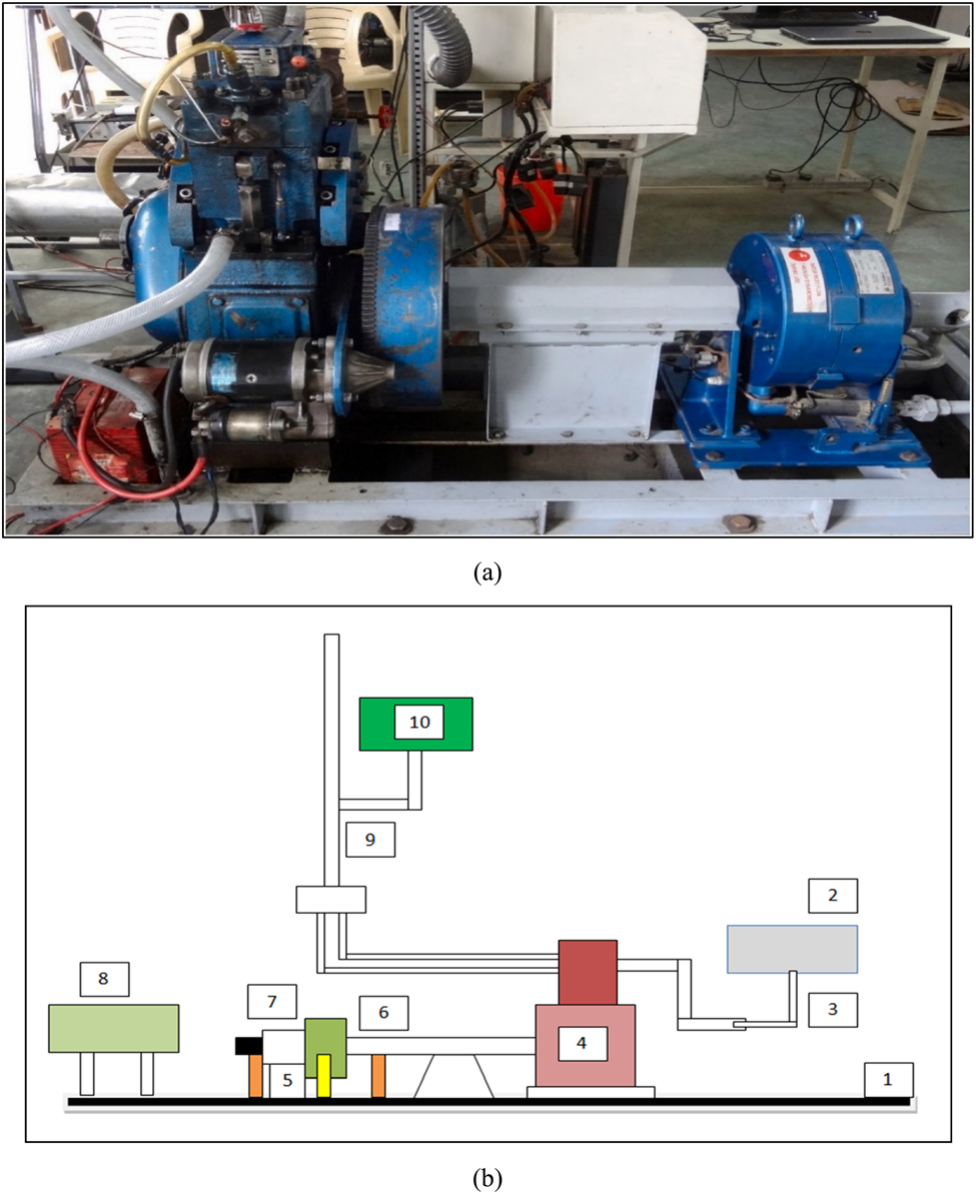
Experimental setup (a) and line diagram (b) of the diesel engine testing arrangement.^20^ (1) Engine base, (2) analyzer for exhaust gas, (3) house of the exhaust gas analyzer, (4) single-cylinder arrangement engine, (5) load cell, (6) dynamometer, (7) tachometer, (8) control system, (9) fuel burette and (10) fuel tank.

The experiments were carried out on a single-cylinder, four-stroke, and water cooled, direct injection diesel engine (Fig. 5). The technical specifications are shown in Table 3. The engine was subjected to modifications into an LHR configuration when it was tested with a nano-emulsified cotton seed biodiesel blend (B20W10Al200). The adjustment involved applying a 200 µm fly ash coating on the cylinder liner, piston crown, and valves, covering them with ash. Following this, tests were done while maintaining the engine speed at 1500 rpm. The engine was then subjected to five incremental load conditions of 0%, 25%, 50%, 75%, and 100%. Emissions were measured using an AVL 444 gas analyzer and AVL 437 smoke meter. A settling chamber was located at the air intake, ensuring that there was no fluctuation in airflow, and thus creating a steady environment. Also, k-type thermocouples were utilized to record the temperature of the exhaust gases. These parameters were monitored by employing a Kistler piezoelectric pressure sensor along with a crank angle encoder placed on the output shaft of the motor, perfectly synchronized with the motion of the engine. The Heaviside step function was combined with an appropriate signal conditioning system to enable data capturing. Throughout testing, a mechanical stirrer was required to ensure that the Al_2_O_3_ nanoparticles remained uniformly dispersed within the fuel blend emulsion. This setup enabled an extensive in-depth examination of the changes in emission under real-time operating conditions in the LHR engine. Meanwhile, for a crankshaft position, the encoder was arranged at the output shaft of the engine. The signal conditioning setup communicates between the piezoelectric pressure sensor and the data acquisition system.^37^

**Table 3:** Technical specifications of the testing engine

Factor	Specifications
**Testing Engine specifications**	
Type of engine	Direct injection (DI) diesel engine
Category	Single cylinder, four stroke
Power	3.5 kW (@1500 ± 50 rpm)
Type of cooling	Water cooled
CR range	12 : 1–18 : 1
Injection variation	0–25°BTDC
Combustion compartment	Semicircular bowl in piston type
Dynamometer	Water cooled with loading unit
Airbox	MS fabricated with orifice meter and manometer (100-0-100)
Fuel reservoir	Volume15 lit with measuring tube (0–450 mL)
Calorimeter	Pipe-in-pipe type
Data attainment software	‘Soft-engine’ engine performance analysis software

**Transmitters, sensors, and indicators**	
Fuel flow reader	DP transmitter, range 0–500 mm WC
Airflow transmitter	Pressure transmitter (−) 250 mm WC
Pressure sensors	Piezo type, range 5000 psi, with low noise cable
Temperature sensors and transmitters	PT100 (RTD) type, range 0–100 °C, output 4–20 mA (4 nos)
	K (ungrounded) type, range 0–1200 °C, output 4–20 mA (2 nos)
Load sensor and indicator	Strain measure-type load cell with digital pointer, range 0–50 kg
Speediness sensor and gauge	Resolution 1°, range (5500 rpm) with TDC pulse
Data acquisition device	NIUSB-6210, 16-bit, 250 kS s^−1^

**Constants in the testing engine**	
Pulse per revolution	360°
No. of cycles	10
Fuel measuring interval	60 s
Speed scanning intervals	2000 ms
Bore × stroke	87.6 mm × 110 mm
Capacity	662 cc
Cavity diameter	2 mm
Dynamometer arm length	18 mm
Linking rod length	235 mm

A special program algorithm was adopted in the data processing unit, where it takes an average of more than 50 uninterrupted cycles to get an effective assessment of the heat release rate, time for the combustion process, *etc.* Under the initial conditions, the testing process started with neat diesel as the test fuel. This step worked as a warm-up step for the engine, and then it was substituted for the nanoparticle-emulsified cotton seed biodiesel fuel. To clean the fuel line and fuel injection system, the engine ran on neat diesel fuel at the end.

### 3.1. Response surface methodology (RSM)

RSM is a robust quantitative and mathematical method applied in experimental design and optimization. Its main goal is to represent the relationship between a number of independent variables and response variables, providing researchers with valuable information on the optimal conditions for a given process.^39^ RSM is particularly handy in trials with complex relationships between variables, where it is difficult to comprehend their combined effect on the system output. The methodology follows a systematic and organized framework, guiding researchers through test design, and then data analysis.^40^ In RSM, the construction of a response surface plays a central role, which is a mathematical model illustrating how the input and output variables are related.

This surface enables investigators to understand the behavior of the system within the experimental area, and thus ascertain the optimal conditions that lead to the desired outcome. RSM facilitates the build-up of proper models, reflecting the intricacies of the system being studied using statistical methods such as regression analysis. RSM is applied in different fields, ranging from engineering and physics to biological sciences, to maximize processes as well as performances. It is possible for researchers to comprehend the performance of a system and successfully identify the most appropriate operating conditions by making systematic changes to the input variables while observing the resulting changes in the response variable. Additionally, RSM is an economical approach as it minimizes the number of experimental runs necessary for solid outcomes.^41^

### 3.2. Machine learning: XGBoost

XGBoost is a powerful and flexible method that has gained popularity in a wide range of disciplines. This method belongs to the ensemble learning family, specifically boosting, which aggregates weak learners to build a strong and accurate prediction model. One unique feature of XGBoost is that it can tackle classification and regression tasks effectively.^42^

The focus on breaking the challenges facing gradient boosting methods, such as over fitting and computational inefficiency, sets XGBoost apart. XGBoost finds a balance between model complexity and accuracy using regularization methods and a new objective function, which consists of both a loss function and a regularized function.^43^ This makes it less prone to over fitting, irrespective of big data. The ‘gradient boosting’ process of this algorithm involves the stepwise construction of decision trees to correct flaws in earlier models, constantly improving the overall predicting capacity. XGBoost further introduces complexity by employing a stronger optimization method, making it particularly well-suited to handle enormous databases containing disparate feature types. Its ability to identify complex data patterns and produce feature importance scores increase the interpretability, while making it applicable in real-world scenarios.^44^

Also, its speed, flexibility, and ability to handle various types of data are all reasons for its use in applications ranging from banking to healthcare. In addition, the integration of XGBoost with Python and other programming languages enables straightforward installation and easy integration with existing ML pipelines. Fundamentally, XGBoost is the algorithm of choice for practitioners seeking a reliable, fast algorithm that performs superbly in predicting accuracy and generalization across a broad variety of datasets.^45^

**Fig. 6:**
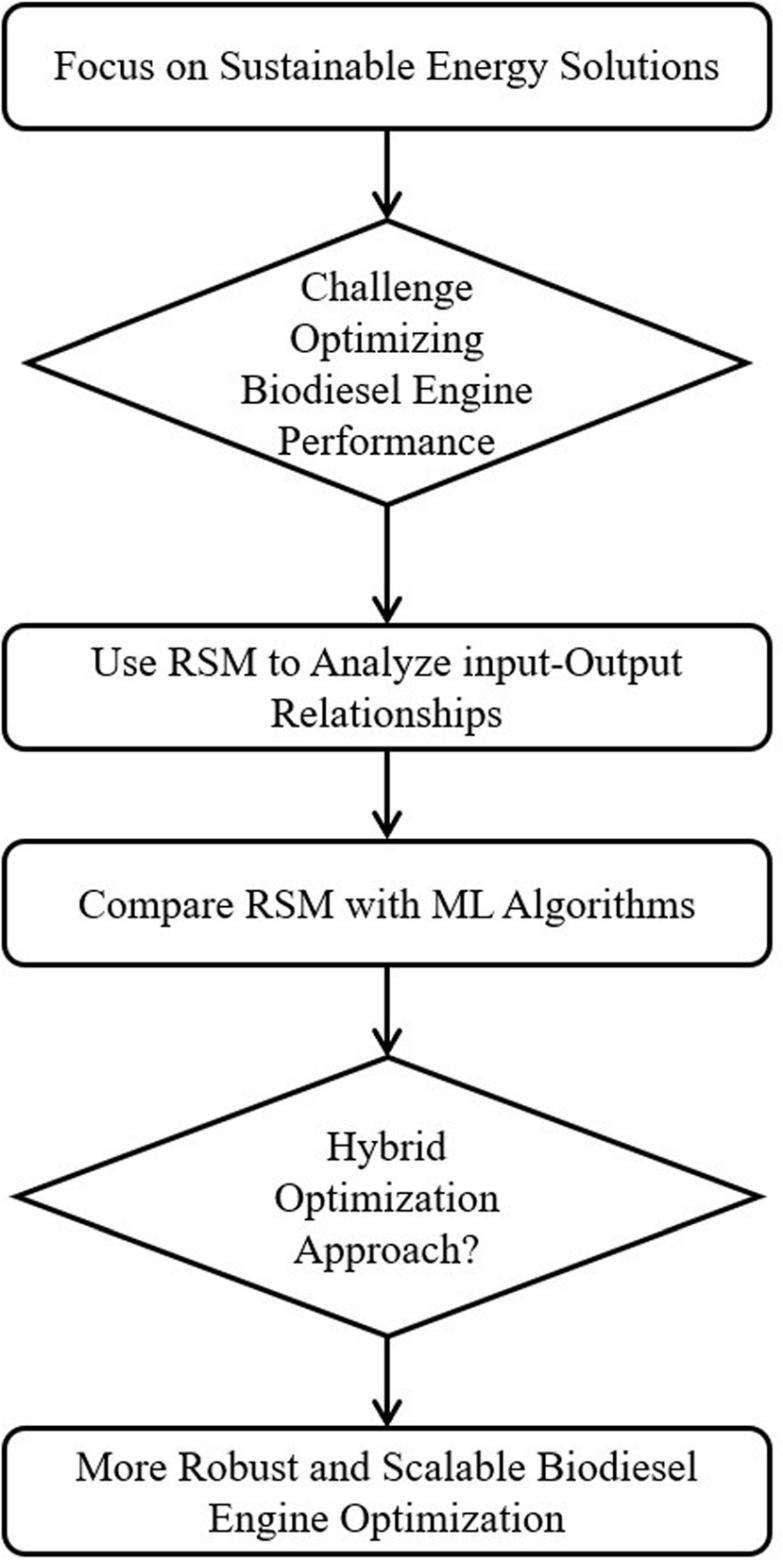
Flowchart for biodiesel engine optimization using RSM and ML.

The overall process flow chart is provided in Fig. 6 for a better understanding.

### 3.3. Interpretable machine learning

SHapley Additive exPlanations (SHAP) is a powerful interpretability method grounded in cooperative game theory, which explains individual predictions of machine learning models. It gives each characteristic a SHAP value, which is a measure of how important it is to the prediction, to ensure that the process is fair and consistent. The main notion comes from Shapley values, which examine each feature as a “player” in a prediction game and figure out its marginal contribution by looking at all possible subsets of features. SHAP makes an additive explanation model by taking the model output and subtracting a baseline (usually the average forecast) to get the total of the feature contributions. SHAP creates a global and local interpretability framework. Globally, it shows which characteristics have the most impact on the model over the whole dataset, and locally, it explains each prediction. TreeSHAP lets you quickly and accurately deal with tree-based models like XGBoost and Random Forest. Force plots, dependency plots, and summary plots are examples of SHAP visualizations that assist stakeholders in understanding how a model works, finding relationships between features, and ensuring that everything is clear. SHAP helps satisfy regulatory requirements in sensitive areas like banking, healthcare, and policy by breaking down black-box models into understandable insights. It also helps with debugging and creating confidence.

## 4. Results and discussion

Various performance and emission characteristics for varying fuel samples at various CR were the subject of the experimental study. The main aim was to determine the way parameters such as BTE, BSFC, HC emissions, CO, NOx, and smoke opacity are affected by the compression ratio, BP, and load.

### 4.1. Net heat release rate (NHR)

**Fig. 7:**
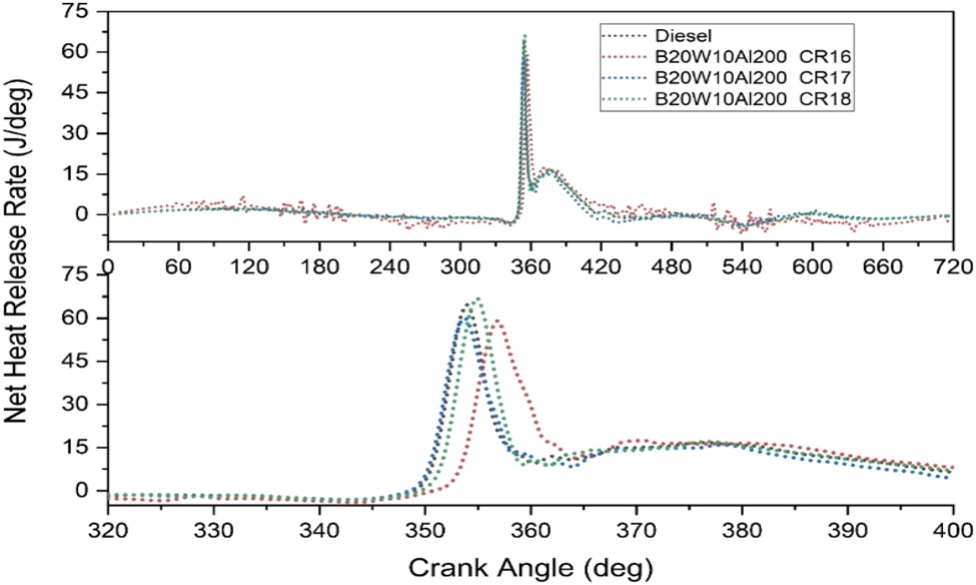
NHR as a function of crank angle for different CR.

The rate at which heat is produced during the combustion process in an engine is measured by the heat release rate (HRR).^46^Fig. 7 shows the NHR rate for various CR at various crank angles. Compared to pure diesel, all experiments show the same NHR trend. Interestingly, the NHR is determined to be the lowest for CR16 and highest for CR18, which is 65 J per °. This is due to the lower ignition temperature brought on by a lower CR.^47^ The air-fuel mixture may not achieve the required temperature for effective combustion with a reduced compression ratio, resulting in delayed and less intense heat release during combustion.

By clearly explaining the concept and utilizing the appropriate terminology, the revised content enhances the understanding of the relationship among the compression ratio, NHR, and combustion process in the engine.

### 4.2. In-cylinder pressure

**Fig. 8:**
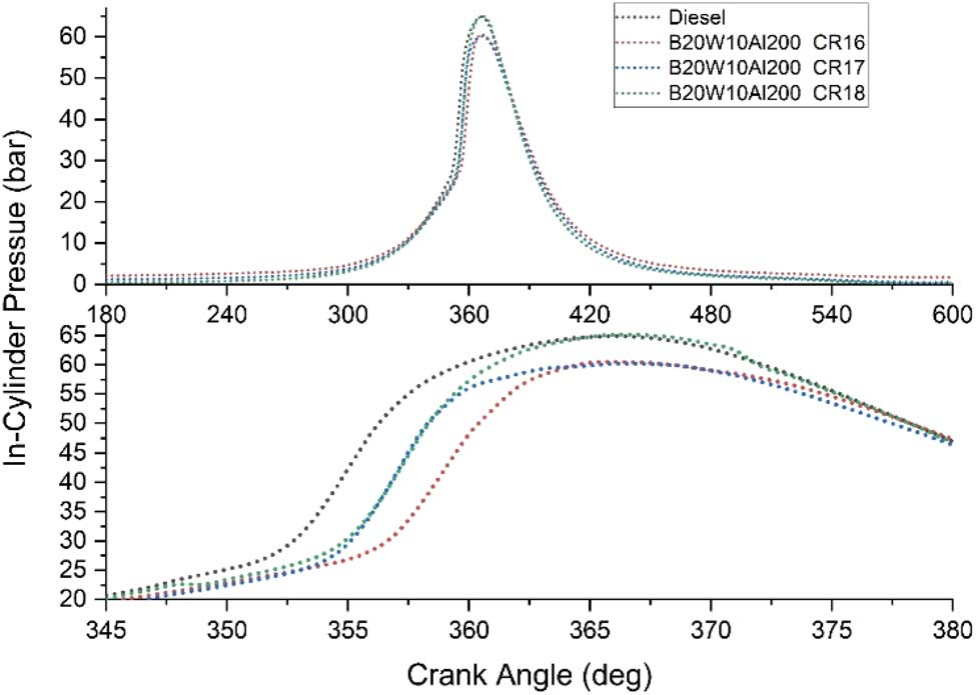
In-cylinder pressure *versus* crank angle for various CR.

Fig. 8 illustrates a consistent pattern observed across all compression ratio (CR) values, including 16, 17, and 18. Notably, the peak in-cylinder pressure is recorded as 64.8 bar, occurring at a crank angle of 364° for a compression ratio of 18.

This finding suggests that irrespective of the specific CR value, the highest in-cylinder pressure is consistently achieved at a similar crank angle. In this case, the compression ratio of 18 yields the maximum peak pressure. The precise crank angle and corresponding in-cylinder pressure provide valuable insights into the combustion process and engine performance.^48^

### 4.3. Brake thermal efficiency

**Fig. 9:**
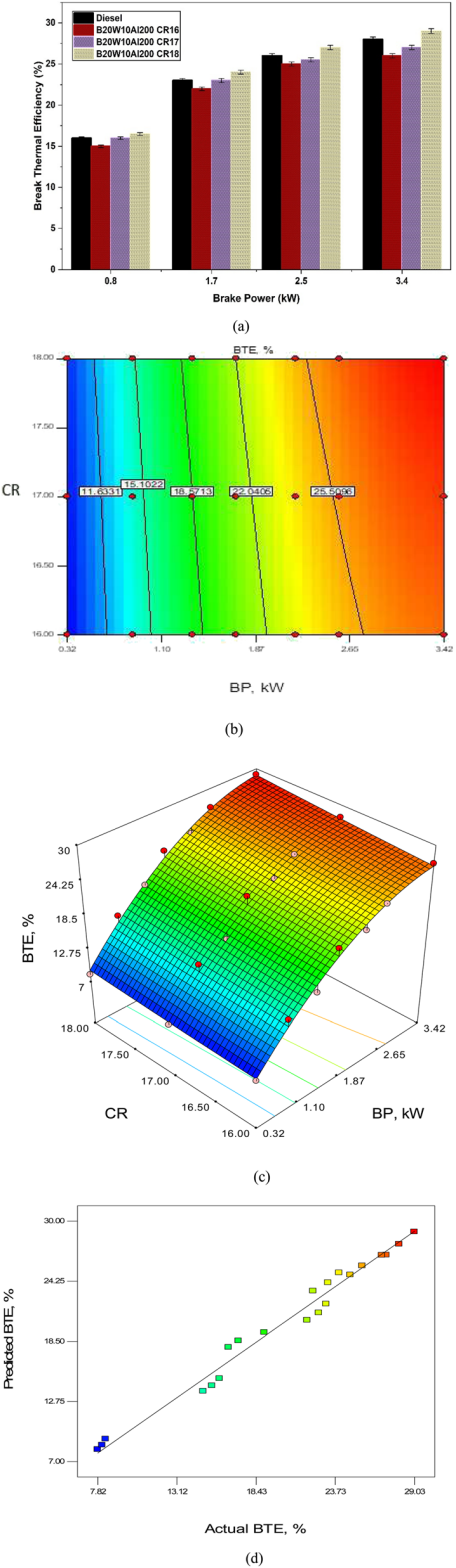
Impact of BP on BTE for different CR: (a) test values, (b) contour plot, (c) surface plot and (d) predicted *vs.* actual values.

An important metric for determining how well fuel is used in an engine is the brake thermal efficiency (BTE).^49^Fig. 9 shows how BP affects BTE, showing how BTE increases gradually as BP increase. Additionally, an increased CR shows an improvement in the BTE of the fuel samples. Among the various CR values tested, B20W10Al200 at CR18 demonstrates the highest BTE compared to CR16 and CR17. This indicates that combining B20W10Al200 fuel and CR18 results in a superior BTE performance. Additionally, implementing a sophisticated heat release rate (HRR) technique at CR18 outperforms the other CR values.^50^ Notably, the BTE achieved at CR18 surpasses the CR16 efficiency by approximately 6.92%. The higher the heat release rates attained when subjected to a CR of 18 leads to a superior BTE than CR of 16 and CR of 17.^51^

The data gathered through lab-based experiments was used to for analysis of variance (ANOVA) to find the link between different data columns. The outcomes of ANOVA are listed in Table 4. The model has an *F*-value of 150.33, indicating that it is statistically significant. A “model *F*-value” of this magnitude arising entirely from noise is only 0.01% likely. Model terms are considered relevant when the value of “Prob > *F*” is less than 0.0500. The significant model terms in this case are *A*, *B*, and *A*^2^. Values greater than 0.1000 indicate that the model terms are unimportant. Model reduction approaches can improve the model if it contains many insignificant terms. The developed model for BTE is given as eqn (1). The model was used to predict values at different engine settings. A comparison of the actual and model-predicted table values is shown in Fig. 9a. It can be observed that the model performed well given that most of the point lies on the best-fit line. The contour plot (Fig. 9b) and surface plot (Fig. 9c) depict that the peak BTE efficiency is achieved at a higher engine load and compression ratio.^29^Fig. 9d represents the predicted *vs.* actual values of BTE, where it can be observed in the graph that both values are very close and approximately linear (Fig. 9d).

1 BTE = 18.395 + 9.15 × BP − 2.04 × CR + 0.198 × BP × CR − 1.96 × BP2 + 0.073 × CR2

**Table 4:** ANOVA of BTE data

Source	Sum of squares	d*F*	Mean of squares	*F*-value	*p*-Value prob > *F*	
Model	839.81	5	167.96	150.33	<0.0001	Significant
*A*-BP	764.06	1	764.06	683.86	<0.0001	
*B*-CR	9.01	1	9.01	8.06	0.0124	
AB	0.52	1	0.52	0.46	0.5060	
*A* ^2^	55.40	1	55.40	49.58	<0.0001	
*B* ^2^	0.025	1	0.025	0.022	0.8836	
Residual	16.76	15	1.12			
Cor total	856.57	20				

### 4.4. Brake specific fuel consumption (BSFC)

**Fig. 10:**
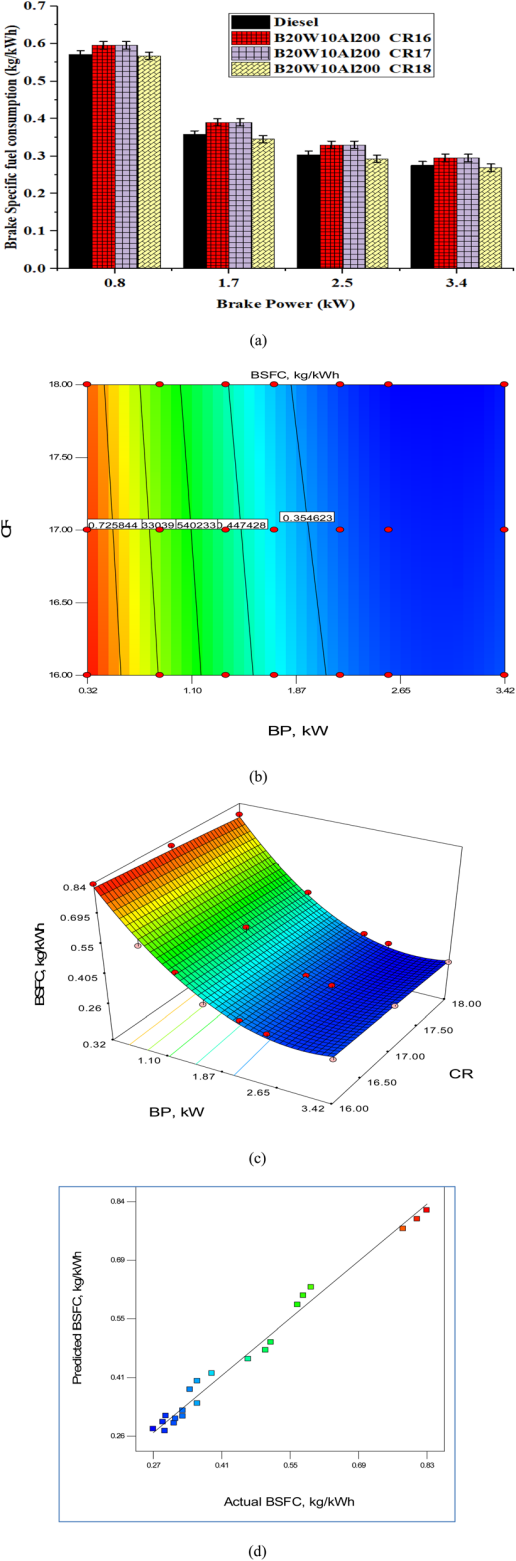
Impact of BP on BSFC for different CR: (a) test values, (b) contour plot, (c) surface plot and (d) predicted *vs.* actual values.

Fig. 10a–d represent BP *vs.* BSFC, BP *vs.* CR (optimum BSFC), 3D representation of BP, BSFC and CR, and predicted *vs.* actual BSFC values, respectively. Interestingly, a similar pattern emerges here as well. The BSFC values for different fuel samples, namely diesel and nano-emulsified fuel at CR16, CR17, and CR18, are recorded as 0.275, 0.305, 0.288, and 0.269 kg kW^−1^ h^−1^, respectively. Notably, the emulsified fuel sample at *CR16* exhibits the highest BSFC among the tested samples. However, as the compression ratio (CR) increases, there is a reduction in BSFC. This finding aligns with the underlying principle of improved combustion rate, which ultimately leads to better fuel efficiency. Particularly, the emulsified fuel sample at the higher compression ratio of *CR18* demonstrates the lowest BSFC compared with the other fuel samples. The observation of lower BSFC values for higher CR values substantiates the notion of enhanced combustion efficiency and improved fuel utilization. This information underscores the importance of increasing the CR to achieve lower BSFC values, thus optimizing the fuel efficiency.

The model *F*-value, a substantial 208.48, emphasizes the robustness of the model, showing an insignificant 0.01% chance of having such a huge value arising due to unpredictability. The results of ANOVA are listed in Table 5. Regarding the model relevance, terms A, B, and A^2^ stand out (Prob > *F* of 0.0500), while values over 0.1000 indicate insignificance. If there are many inconsequential model terms (except those required for hierarchy), a model reduction could improve its effectiveness. Std. Dev., *R*^2^ (0.9858), and Adeq. Precision (41.031) constitute key metrics that add to the reliability of the model, and the significant “Pred *R*-Squared” (0.9735) fits nicely with the “Adj *R*-Squared” (0.9811), emphasizing the predictive accuracy. The Adeq. Precision ratio, which is more than 4, emphasizes a favorable signal-to-noise ratio, enabling effective design space exploration and enhancing the practical usability of the model. The mathematical model developed using ANOVA is given in eqn (2). The model was used to forecast values at various engine settings. It can be seen that the model performed well because the majority of the points are on the best-fit line. The lowest BSFC was at a higher engine load and compression ratio, as shown by the contour plot (Fig. 10b) and the surface plot (Fig. 10c). Fig. 10d represents the predicted *vs.* actual values of BSFC and it can be observed that both values are very close and approximately linear, as shown in the graph. Hence, the model is more suitable for future studies.

2 BSFC = 1.0211 − 0.474 × BP + 0.0128 × CR + 0.0022 × BP × CR + 0.074 × BP2 − 0.00107 × CR2

**Table 5:** ANOVA of BSFC data

Source	Sum of squares	d*F*	Mean of squares	*F*-value	*p*-Value prob > *F*	
Model	0.64	5	0.13	208.48	<0.0001	Significant
*A*-BP	0.52	1	0.52	842.73	<0.0001	
*B*-CR	5.315E-003	1	5.315E-003	8.59	0.0103	
AB	6.191E-005	1	6.191E-005	0.10	0.7561	
*A* ^2^	0.11	1	0.11	170.51	<0.0001	
*B* ^2^	5.357E-006	1	5.357E-006	8.661E-003	0.9271	

### 4.5. Unburned hydrocarbons (HC)

**Fig. 11:**
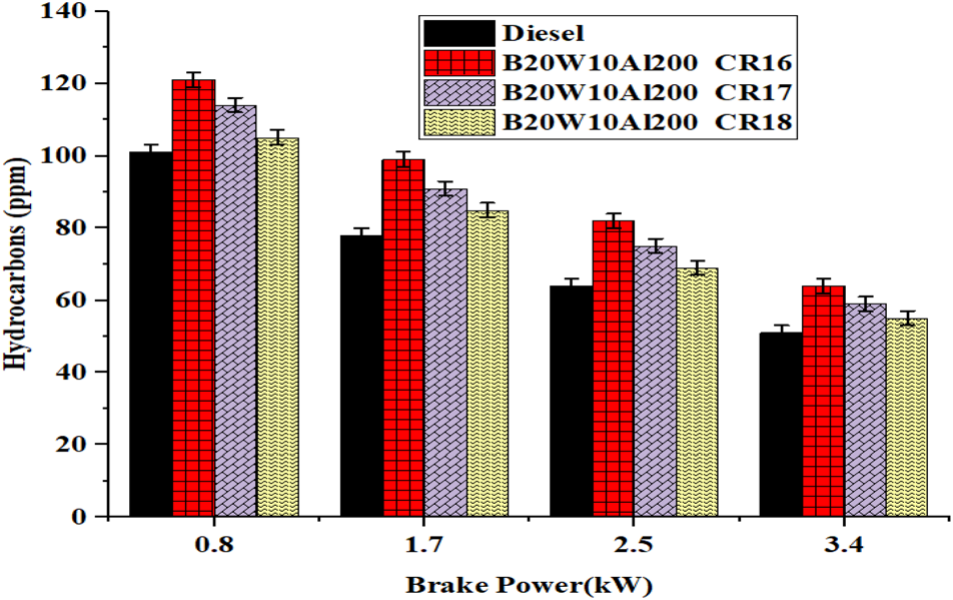
Influence of brake power on HC emission formation.

Hydrocarbon emissions are critical parameters of incomplete combustion and are sensitive to the engine load, fuel type, and compression ratio (CR). In this case, the HC emissions appeared to decline with an increase in brake power across all fuel samples, indicating an improved combustion efficiency at higher loads because of the higher in-cylinder temperatures. Fig. 11 illustrates the impact of BP on the HC emissions.

Fig. 11 illustrates the impact of brake power (BP) on HC emissions. Among the tested fuels, diesel had the lowest HC emissions of 51 ppm due to its good combustion property. The emulsified cotton seed biodiesel blends showed slightly higher HC emissions of 64 ppm at CR16, 59 ppm at CR17 and 55 ppm at CR18. Especially, CR16 demonstrated 25.5% more HC emissions than diesel, explaining the reason for the poor atomization and lower in-cylinder temperatures at CR16, which prolonged the ignition delay and led to unburned fuel. As the CR increased, the thermodynamic conditions became more favorable with regard to fuel atomization and enhancement of flame front propagation. The nanoparticles of aluminum oxide enhanced micro-explosions and burned more uncombusted hydrocarbons. This was most effective at a CR of 18. The results show that exhaust HC emissions can be reduced by optimizing the CR and using nano-emulsified biodiesel to promote more complete combustion. These observations highlight the importance of optimizing the CR to achieve a higher burning rate and minimize HC emissions. Hydrocarbon outputs from the biodiesel blend arise from the aerobic biofuel section, facilitating a more thorough burning of chemical forms. The breathable air promotes enhanced oxidative damage of HCs, specifically at the higher levels accomplished in the LHR engine arrangement.

### 4.6. Carbon monoxide (CO)

**Fig. 12:**
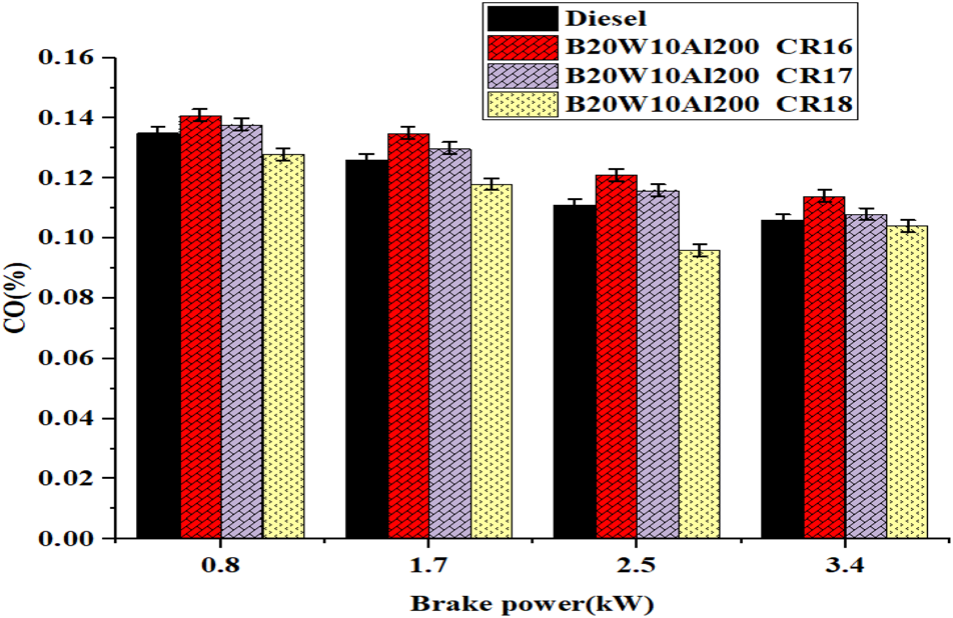
Influence of brake power on carbon monoxide formation.

Fig. 12 reinforces the importance of considering the impact of load and BP on CO formation during the combustion process. CO formation reaches its lowest point at 35.75% of the maximum brake pressure for the tested fuel. Interestingly, CO formation decreases as the load increases due to the improved combustion efficiency. Among the different CR tested, CR18 consistently exhibits the lowest CO emissions. For instance, the CO emissions for the diesel and nano-emulsified fuel at CR16, CR17, and CR18 were recorded to be 0.10%, 0.11%, 0.10%, and 0.09%, respectively. These findings highlight the significance of optimizing the compression ratio to minimize the CO emissions and improve the combustion efficiency. A reduced quantity of CO was observed. Effective consumption is achieved through enhanced the swirling and squished movement of air with an increase in oxygen in the blends within a low heat rejection cylinder.

### 4.7. Nitrogen oxide (NOx)

**Fig. 13:**
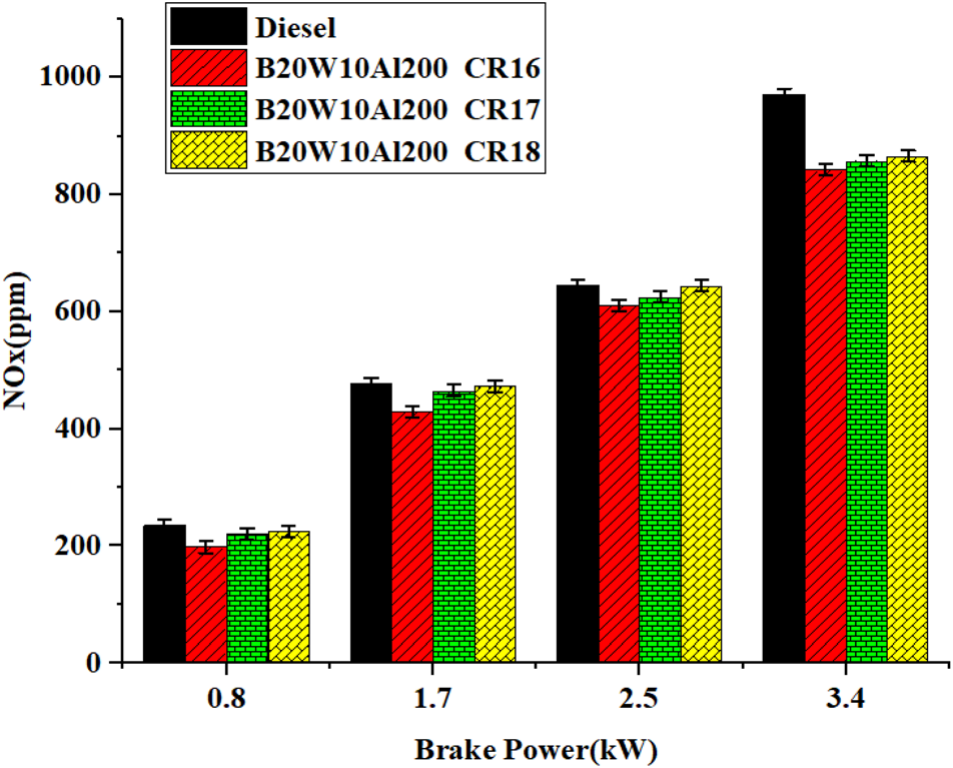
Influence of brake power on NOx formation.

Fig. 13 shows the correlation between braking power (BP) and NOx emissions at various CR. Due to the high ignition temperatures experienced during combustion, NOx emissions are primarily produced. An emulsion fuel was used in the experiment, which reduced the ignition temperature.

In contrast to the other tested samples, the results showed that diesel fuel had the highest NOx emissions. Notably, the NOx emissions at CR16 were 13.5% lower than diesel emissions. This can be explained by the lower ignition temperature brought on by the lower CR of 16. NOx emissions diminish under every load scenario as the perfect level increases. When the combustion chamber engines are coated with the LHR material, oxides of nitrogen are diminished due to the smaller amount of oxygen and decreased flame temperature.

### 4.8. Smoke opacity

**Fig. 14:**
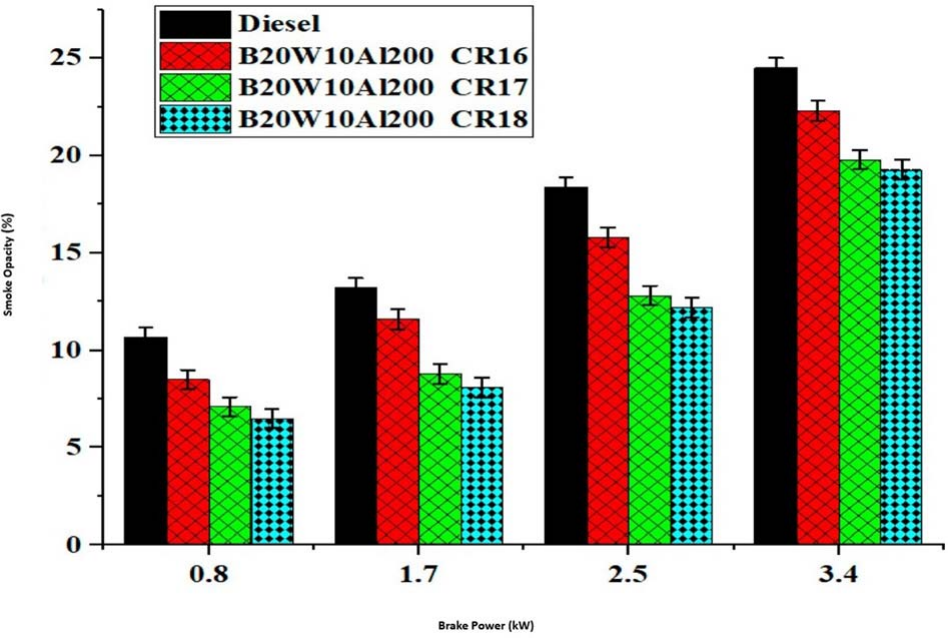
Influence of brake power on smoke formation.

Smoke opacity in a diesel engine is the measure of how much the exhaust blocks light, indicating the amount of soot and particulate emissions. Due to the reduced oxygen availability, the smoke opacity tends to increase in dual-fuel mode. Fig. 14 illustrates the impact of brake power (BP) on the smoke opacity. Among the different CR, CR18 exhibited the lowest smoke opacity due to the sophisticated temperature achieved with a higher CR. Specifically, at CR18, the smoke opacity was recorded as 24.31% less than diesel fuel. This significant reduction can be attributed to the optimized temperature conditions resulting from the higher compression ratio. The higher CR facilitates an improved combustion efficiency, leading to a decrease in smoke opacity.^45^

These findings highlight the importance of selecting an appropriate compression ratio to minimize the smoke opacity in dual-fuel mode by considering the influence of BP and optimizing the CR.^45^

## 5. Optimization with RSM

The present study employed the RSM technique to optimize the compression ratio and brake power to establish the best combination to provide the maximum efficiency, and the lowest possible fuel consumption and emission. The design matrix used in the present study was prepared using a historical data approach, as shown in Table 6.

**Table 6:** Design matrix

Run	BP, kW	CR	BTE, %	BsFC, kg kW^−1^ h^−1^	NOx, ppm	HC, ppm	CO, %	Smoke OP. %
1	0.32	16.00	7.82	0.834	95	146	0.168	4.9
2	0.86	16.00	14.89	0.595	198	121	0.141	8.5
3	1.35	16.00	16.58	0.512	245	105	0.138	9.3
4	1.71	16.00	21.85	0.39	429	99	0.135	11.6
5	2.20	16.00	22.25	0.36	508	88	0.127	12.3
6	2.56	16.00	24.74	0.33	610	82	0.121	15.8
7	3.42	16.00	27.15	0.295	842	64	0.114	22.3
8	0.32	17.00	8.12	0.814	101	128	0.164	3.6
9	0.86	17.00	15.48	0.579	220	114	0.138	7.1
10	1.35	17.00	17.25	0.501	265	101	0.133	8.2
11	1.71	17.00	22.63	0.36	465	91	0.13	8.8
12	2.20	17.00	23.25	0.33	535	83	0.124	11.4
13	2.56	17.00	25.54	0.312	625	75	0.116	12.8
14	3.42	17.00	28.01	0.289	858	59	0.108	19.8
15	0.32	18.00	8.35	0.785	105	115	0.16	3.3
16	0.86	18.00	15.98	0.567	224	105	0.128	6.5
17	1.35	18.00	18.98	0.465	321	82	0.125	7.5
18	1.71	18.00	23.12	0.345	498	85	0.118	8.1
19	2.20	18.00	23.99	0.315	565	74	0.12	10.4
20	2.56	18.00	26.85	0.293	644	69	0.096	12.2
21	3.42	18.00	29.03	0.269	866	55	0.104	19.3

### 5.1. Optimization on a desirability basis

**Fig. 15:**
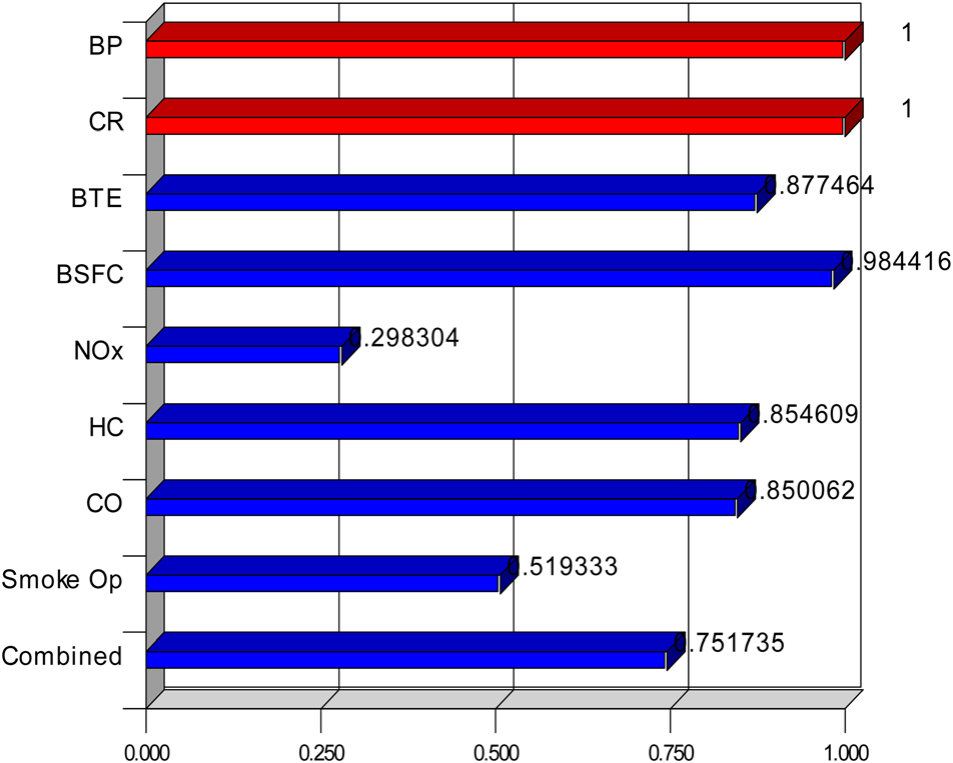
Desirability plot.

The established method of desirability was used in the present study for parametric optimization. The BTE was desired to be as high as possible, while the remaining parameters were desired to be as low as possible. A trade-off analysis was conducted using Design-Expert software. The results are shown in Table 7. The desirability plot is shown in Fig. 15.

**Table 7:** Optimized control factors and response variables

Control factors		Response variables						
BP	CR	BTE	BSFC	NOx	HC	CO	Smoke Op	Desirability
2.49	18	26.43	0.2778	636	68.23	0.106	12.43	0.751

### 5.2. Model prediction with XGBoost

Advanced ML techniques are increasingly important in optimizing the engine performance, while minimizing environmental effects. This study investigates the implementation of XGBoost, an ensemble learning algorithm, for predicting and modeling engine performance metrics. This study is focused on using the compression ratio and braking power as predictors to establish connections with key engine response characteristics such as brake thermal efficiency, braking specific fuel consumption (BSFC), NOx emissions, CO, HC emissions, and smoke.

The methodology starts with the data acquisition and preprocessing of a dataset comprising different CR and braking power values. These are crucial inputs as they represent the inherent characteristics of the engine under different operating conditions. The response variables like BTE, BSFC, NOx emissions, CO, HC emissions, and smoke levels are closely monitored and measured to capture the diverse engine performances and emissions characteristics. The data was randomly divided in a 70 : 30 ratio for training and testing. The grid search-based hyperparameter optimization was employed in this study. The hyperparameter range and optimized values are listed in Table 9.

XG Boost, which has proven ability to cope with high-order interactions and various data sources, is then used to develop prediction models. The approach builds an ensemble of decision trees iteratively and learns from the patterns of the training data for predicting the response variables accurately. Regularization methods in XGBoost reduce the risk of overfitting, ensuring strong generalization to new, unseen data. Validation and optimization of XGBoost models are necessary steps within the research method. The data is split into training and test sets, allowing the model performance to be measured against independent information. Increasing the predictive power of the model, hyper parameter tuning and cross-validation strategies enhance its reliability and accuracy in modeling the complex interactions among CR, braking power, and engine reaction variables.

**Fig. 16:**
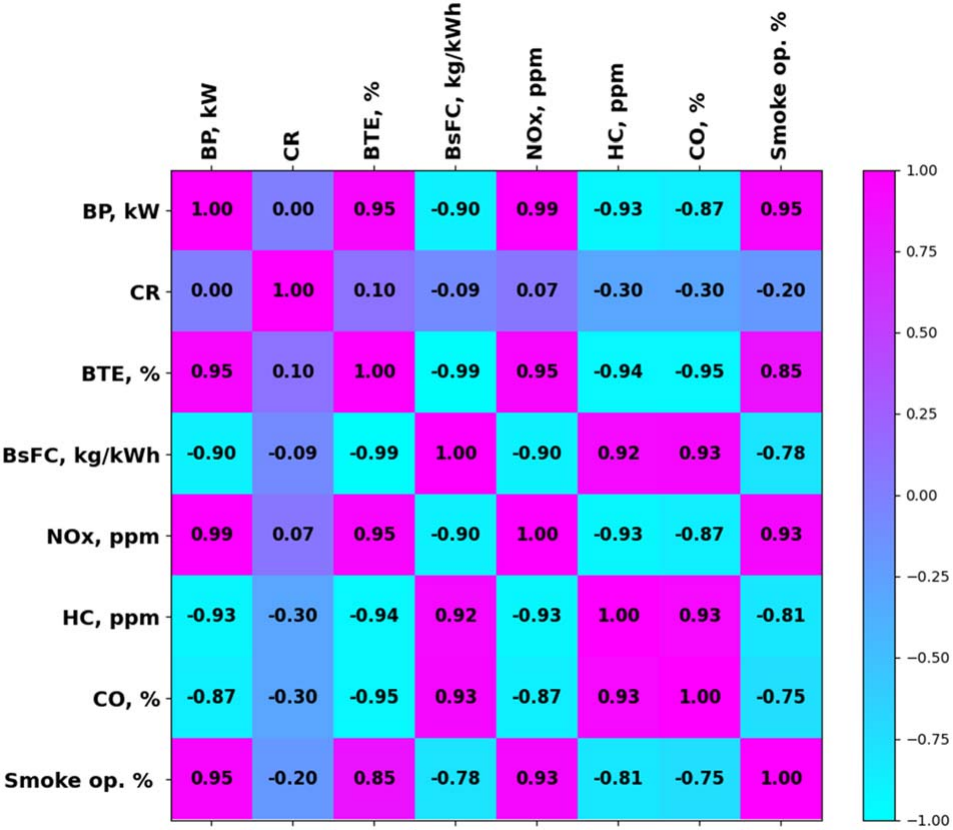
Correlation heat map.

The correlations between data columns are shown in Fig. 16 as a correlation heat map. After the model was created, it was used for prediction. The statistical measures in Table 8 provide a detailed evaluation of how well a predictive model performs during the training and testing phases, highlighting important engine response features. *R*^2^, mean squared error (MSE), and mean absolute percentage error (MAPE) are some of the measures used to gain more detailed insight into the accuracy of the model and its prediction ability.

**Table 8:** Statistical evaluations of the model prediction results

	Train *R*^2^	Train MSE	Train MAPE	Test *R*^2^	Test MSE	Test MAPE
BTE	1	0.0004	0.082	0.9288	0.7299	3.4051
BSFC	0.9999	0.0000009	0.21458	0.9931	0.0003	2.1951
NOx	0.9999	0.3919	0.2259	0.9848	500.34	9.365
HC	0.9989	0.352331	0.5066	0.9026	103.46	9.317
CO	0.9517	0.00001	2.0451	0.9137	0.00002	3.745
Smoke Op	0.996	0.06999	2.314	0.942	2.38	7.674

**Table 9:** Range of hyperparameters and optimized values

Hyperparameter	Search range	BTE	BSFC	NOx	HC	CO	Smoke OP.
Learning_rate	[0.01, 0.05, 0.1]	0.1	0.5	0.1	0.1	0.5	0.05
Max_depth	[3, 4, 5]	3	5	5	3	4	3
n_estimators	[50, 100, 150]	150	150	150	100	50	150
Subsample	[0.7, 0.9, 1.0]	0.9	0.7	0.7	0.7	1	0.7

**Fig. 17:**
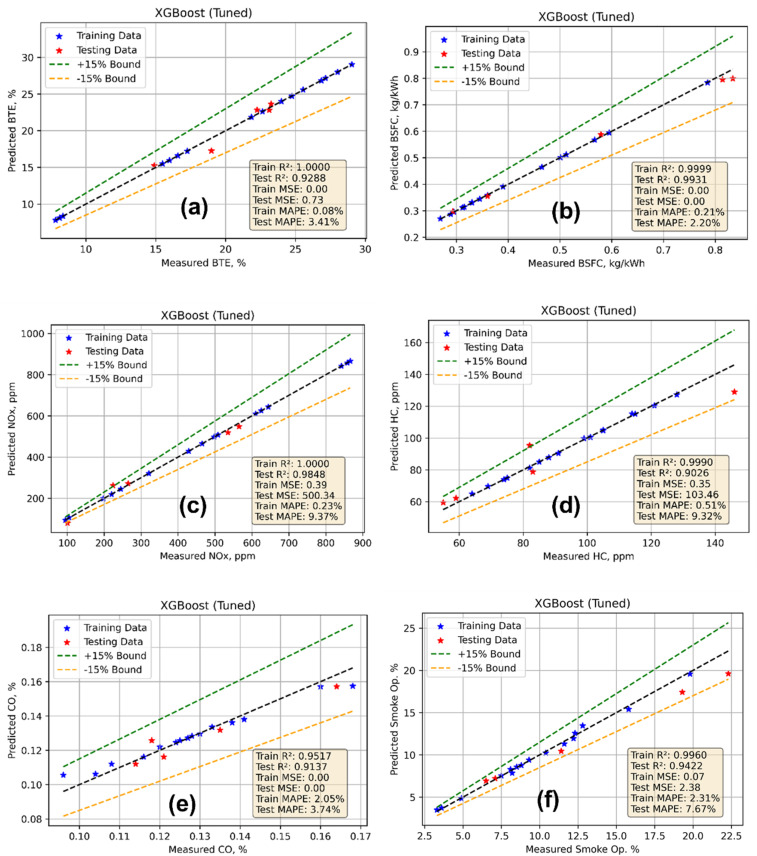
Model prediction *vs.* actual data. (a) BTE, (b) BSFC, (c) NOx, (d) HC, (e) CO and (f) smoke opacity.

Table 8 and Fig. 17 depict the performance of the XGB models applied to predict BTE, BSFC, NOx, HC, CO, and SO using key input features. The model does a great job at training for BTE (Fig. 17a), with *R*^2^ = 1 and MSE = 0.0004, which means it fits perfectly. The excellent generalization and ability of the model to capture the changes in thermal efficiency across a range of CR and BP circumstances are confirmed by the test *R*^2^ of 0.9288, MSE of 0.7299, and MAPE of 3.41%. The model has *R*^2^ values of 0.9999 (train) and 0.9931 (test), which are almost excellent for BSFC (Fig. 17b). The MSE values are quite low, and the test MAPE of 2.20% shows that the predictions are very accurate. This shows that XGB does a good job of modelling BSFC, which is load sensitive, particularly when the fuel-air conditions are different. The *R*^2^ values for the train and test sets for NOx are 0.9999 and 0.9848, respectively, as shown in Fig. 17c and Table 8. The test MSE is also quite high at 500.34, which suggests that the results may not always be accurate given that NOx generation is nonlinear. However, a test MAPE of 9.37% is acceptable given the complicated thermal NOx dynamics. The train *R*^2^ = 0.9989 and test *R*^2^ = 0.9026 in the HC model show that the model learned well (Fig. 17d). However, the test MAPE of 9.32% shows that there was slightly more error, perhaps because of combustion instability at lower BP and emissions during cold starts. The train and test *R*^2^ scores for the CO model are 0.9517 and 0.9137, respectively (Fig. 17e). The MSE values are quite low, while the test MAPE is 3.75%. Although CO depends on the intensity of the mixture and the amount of oxygen available, these findings imply that the CO predictions are consistent. Lastly, the SO model gives *R*^2^ values of 0.996 for the training set and 0.942 for the test set, as well as a test MSE of 2.38 and a test MAPE of 7.67% (Fig. 17f). These numbers show that XGB does a good job of capturing how soot moves when there is a load and when the CR changes. Overall, all the models train well and make good predictions, with test *R*^2^ values more than 0.90 in all situations. This shows that XGB and the modified hyperparameters are good at modelling complicated engine behaviour with high accuracy.

### 5.3. Interpretation of models

**Fig. 18:**
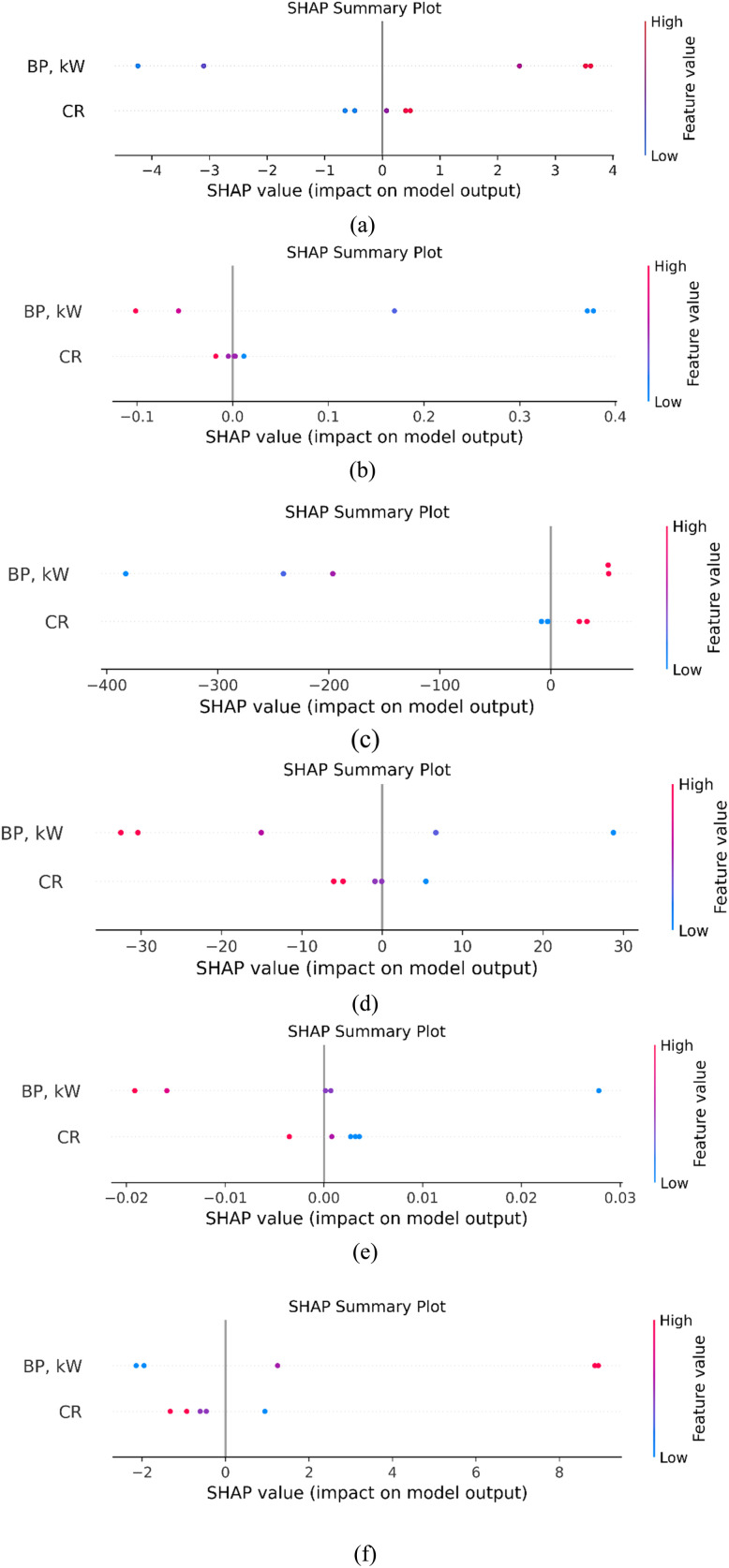
SHAP values for feature analysis for (a) BTE, (b) BSFC, (c) NOx, (d) HC, (e) CO and (f) smoke opacity.

Fig. 18 illustrates the SHAP summary plots for the six XGBoost-based machine learning models developed to predict various engine performance and emission parameters, namely BTE, BSFC, NOx, HC, CO, and smoke opacity. Each subplot (Fig. 18a–f) shows how the ‘output’ of the model is affected by the input characteristics of brake power (BP, kW) and compression ratio (CR), respectively. The colour gradients show feature values from low (blue) to high (red). Fig. 18a (BTE) shows that both CR and BP have a big effect on the output, albeit in different ways. Larger BP values (pink/red) push the BTE forecasts in a good way, which means that larger loads will be more efficient. Conversely, lower CR values tend to decrease the BTE, which means that a greater compression ratio makes the engine more thermodynamically efficient. Fig. 18b (BSFC) shows a tight cluster around zero, with small positive SHAP effects from higher CR and negative effects from lower BP. This means that BSFC becomes better (decreases) as the BP increases and worse (increases) when the CR increases. This is a balance that is very important for getting the best fuel economy. Fig. 18c (NOx) demonstrates that CR has a substantial negative effect on NOx emissions, and higher CR values drive the forecast down. BP also contributes but marginally. This behaviour is consistent with thermodynamic trends, which indicates that increased CR lowers NOx generation given that combustion lasts for a shorter time.

Fig. 18d (HC) shows that decreasing the BP greatly lowers HC emissions, whereas CR has a more random effect. The trend shows that there is incomplete combustion at lower loads, which increases the amount of unburned hydrocarbons. Fig. 18e (CO) indicates that both characteristics have a SHAP effect that is close to zero. This means that the model thinks these features are not very good at predicting CO. This might be because CO is sensitive to other parameters that affect combustion, including the temperature and the air-fuel ratio, which are not included in this narrow collection of features. Lastly, Fig. 18f (smoke opacity) shows that greater BP levels make the smoke levels increases, whereas higher CR levels make them decrease slightly. This is consistent with what happens in real life, where a higher load produces soot, while a high CR makes combustion more complete. These SHAP charts make the XGBoost models easier to understand, confirm the engine performance patterns, and build confidence in the models.

### 5.4. Implications of this study

This study has extensive implications outside of the laboratory, especially in the field of internal combustion engine technology and alternative energy sources. This research presents informative data with extensive implications through the observation of complex relationships among variations in compression ratio, emulsified fuel mixtures, and nano-Al_2_O_3_ additive in a fly ash-coated LHR engine. The RSM recognized ideal conditions along with the ability of XGBoost to perform predictive modeling, thus presenting researchers and engineers with a road map for developing and operating more environmentally friendly internal combustion engines that are more efficient. One significant implication is that there might be the possibility to reduce emissions, something that is of significant concern in the current age of environmentalism. The findings of this study, which indicate the complementary action of nano-Al_2_O_3_ additives and emulsified fuels, suggest an effective way to minimize harmful emissions such as CO and NOx. This has a direct implication in compliance with stringent environmental regulations and creating cleaner, greener modes of transport.

Furthermore, this research propels fuel technology by proving the suitability of emulsified fuels with nano-Al_2_O_3_ additive to enhance combustion characteristics to a high degree. This development could pave the way to devising fuel blends that are both environmentally friendly and cost-effective. These findings can totally revolutionize the operation of machines and vehicles by allowing industries and governments to make informed choices that will determine the future of energy systems and transportation. The improvements in methodology brought by the research in applying advanced optimization methods, such as RSM and XGBoost modeling, have broader implications for experimental design and data analysis. The capability of these techniques to effectively search the complex parameter space of fuel-supplied engines with emulsified fuels sets a benchmark for subsequent research studies seeking to optimize complex systems.

The approaches used in this work may be crucial in tackling issues in various scientific and technical fields as the need for sustainable energy solutions grows. The results of this study can essentially be applied to realize methodological breakthroughs, technological innovation, and environmental stewardship. According to this research, emulsified fuels and nano-Al_2_O_3_ additives are potential components in the continuing effort to help society move to cleaner and more efficient energy sources in the quest for sustainable internal combustion engine technology.

## 6. Conclusions, limitations and future scope

### 6.1. Conclusions

This research investigates the maximization of CR of a fly ash-coated LHR diesel engine operated on a nano-Al_2_O_3_ emulsified cotton seed biodiesel blend (B20W10Al200). The experiment was carried out on three compression ratios of CR16, CR17, and CR18. Among them, CR18 exhibited the best performance with the highest BTE of 29.03% and the lowest BSFC of 0.269 kg kW^−1^ h^−1^. This enhancement is due to the improved combustion efficiency from the higher in-cylinder temperature and efficient atomization from the nano-Al_2_O_3_ additive. The emission analysis showed a significant decrease in CO (0.104%) and smoke opacity (19.3%) at CR18, but NOx emissions were drastically lower at CR16 because of the lower combustion temperatures. The HC emissions decreased progressively with an increase in CR, which shows improved combustion completeness. To confirm and simulate the engine performance, XGBoost machine learning was utilized, which resulted in high prediction accuracy with *R*^2^ values over 0.90 for all parameters. The XGBoost model reliably captured nonlinear trends and showed low prediction errors (MAPE < 10%). RSM was also utilized to carry out multi-objective optimization, where it sought to maximize the BTE and minimize emissions and BSFC. The best conditions were found to be a brake power of 2.49 kW and CR18 with a composite desirability of 0.751.

Overall, the incorporation of nano-biodiesel blends in thermal barrier coatings and intelligent modeling presents a promising route toward increasing the efficiency and minimizing environmental effects in compression ignition engines, moving toward sustainable engine technology.

### 6.2. Limitations

A few limitations should be noted although this work provides useful data on the interaction of emulsified fuels, nano-Al_2_O_3_ additives, and changes in compression ratio in a fly ash-coated LHR engine. It is possible that the controlled laboratory environment in which the testing and analysis were conducted is not fully representative of the varying dynamic conditions engines face in practical use. Additionally, this research focuses on a few specific factors, and the nature of the coating with fly ash, the nano-Al_2_O_3_ concentration, and the specific emulsified fuel composition can all influence the results. Many variables make the combustion processes in internal combustion engines complex, and while efforts have been made to uncover and understand the key factors, there could be others.

### 6.3. Future scope

Future studies can build upon and improve our understanding of emulsified fuel-supplemented engines using nano-Al_2_O_3_ additives by addressing the limitations that have been discovered.

• A more complete picture of the possible trade-offs and synergies in engine performance may be obtained by integrating other factors such as various coating materials, emulsification processes, and types of nanoparticle.

• Broadening the scope of this study to encompass diverse engine categories and operational circumstances, engines using nano-Al_2_O_3_ additives and emulsified fuels may also be conducted to solve engine wear and maintenance issues.

• Advanced computer models and simulations might be used to supplement the experimental results, enabling a more thorough examination of optimization techniques and combustion processes.

## Conflicts of interest

The authors declare no competing interests.

## Nomenclature

Al_2_O_3_: Aluminum oxide
BTE: Brake thermal efficiency
BSFC: Brake specific fuel consumption
BP: Brake power
CO: Carbon monoxide
CO_2_: Carbon dioxide
CR: Crank angle
HC: Hydrocarbon
CR16: Compression ratio of 16
LHR: Low heat rejection
RSM: Response surface methodology
SEM: Scanning electron microscopy
MAPE: Mean absolute percentage error
MSE: Mean squared error
ML: Machine learning
NOx: Nitrogen emissions
HRR: Heat release rate
NHR: Net heat release rate
Ppm: Parts per minute
XG: Extreme gradient boosting

## Data Availability

All data generated or analyzed during this study are included in this manuscript.
